# Application of Levy flight-based harmony search algorithm for the flexible job shop scheduling

**DOI:** 10.1038/s41598-025-01255-0

**Published:** 2025-05-24

**Authors:** Jun Li, Yang Zhou

**Affiliations:** 1 College of Computer Science & Engineering, Northwest Normal University, Lanzhou, 730070 China; 2 School of Science and Engineering, Lanzhou Modern Vocational College, Lanzhou, 730300 China

**Keywords:** Levy flight, Harmony search, Optimization, Flexible job shop scheduling, Engineering, Computer science

## Abstract

The Flexible Job Shop Scheduling Problem (FJSP) is an extension of the classical job shop scheduling problem, which is characterized by the fact that each process can be processed on multiple candidate machines, and needs to solve the two subproblems of machine allocation and process sequencing simultaneously. Since FJSP is an NP-hard problem, its complexity and multi-objective characteristics make the traditional exact methods inefficient. At the same time, the existing intelligent optimization algorithms are prone to falling into local optimums, which makes it difficult to balance global exploration and local exploitation capabilities. To this end, this study proposed a Levy flight-based Harmony Search algorithm (LHS), which effectively avoids premature convergence by dynamically and adaptively adjusting the Harmony Memory Considering Rate (HMCR), the probability of Pitch Adjusting Rate (PAR), and the arbitrary distance Bandwidth(BW), and by introducing a Levy flight mechanism to perturb the parameters to broaden the search space and enhance the diversity of the population. We validate the experiments using 8 × 8, 10 × 10, and 10 benchmark instances proposed by Brandimarte in the literature, and the experimental results show that the Harmony search algorithm based on Levy flight outperforms the other comparative algorithms in terms of the solution quality and the convergence speed, demonstrating the effectiveness of its solution for FJSP.

## Introduction

Manufacturing, as the pillar of the national economy, plays a significant role in economic growth and improving living standards^1^. The strategy of intelligent manufacturing is of great importance to the development of the manufacturing industry. The core idea of intelligent production in manufacturing is to provide a flexible, efficient, and green production model, aiming to improve production efficiency through the deep integration of manufacturing and information technology^2^. Scheduling optimization plays a crucial role in both manufacturing and service industries. The Job Shop Scheduling (JSP) problem, as a typical scheduling optimization problem, has been widely studied in the field of manufacturing systems^3^. Among them, the Flexible Job Shop Scheduling (FJSP) problem is a subclass of the classic JSP^4^. In FJSP, an operation can be processed on one or more candidate machines, making FJSP more complex than JSP^5^.

With the development of mathematics and computer information technology, FJSP has been studied since 1990 when Brucker and Schlie began researching it using polynomial algorithms. Since then, many different methods and algorithms for solving FJSP have emerged, mainly divided into two categories: exact techniques^6^ and approximation techniques^7^. Exact techniques tend to have low solution efficiency due to various constraints^8^, while approximation techniques are widely studied and applied due to their higher solution efficiency^9^. Chaudhry and Khan^10^ conducted a comprehensive survey on the development and solution techniques of FJSP from 1990 to 2014, covering various publications (including books, conference papers, journal articles, and reports). The survey found that 59% of the publications employed hybrid intelligent technologies or evolutionary algorithms to solve FJSP. Solving FJSP typically involves considering two sub-problems: the routing subproblem (machine allocation)^11^ and the scheduling subproblem (operation sequencing). Common intelligent optimization algorithms used to solve FJSP include GA (Genetic Algorithm)^12^, PSO (Particle Swarm Optimization)^13^, ABC (Artificial Bee Colony)^14^, and Artificial Bee Colony (ABC) algorithm^15^.

The FJSP problem is difficult for traditional exact algorithms (e.g., branch-and-bound methods) to cope with the demand of solving large-scale instances due to its NP-hard nature and the dual complexity of machine assignment and process sequencing. Optimization algorithms (e.g., meta-heuristics) are able to find high-quality approximate solutions in a reasonable time by simulating natural phenomena or group intelligence behaviors, and have become the mainstream methods for solving FJSP.

Metaheuristics are a general class of algorithmic frameworks for solving complex optimization problems by simulating natural phenomena or biological behaviors to find an approximate optimal solution to the optimization problem. The core of metaheuristics lies in striking a balance between global and local search in order to avoid falling into local optimal solutions while improving the convergence speed and quality of the algorithm. In recent years, with the increasing complexity of optimization problems, metaheuristics have been used to find approximate optimal solutions to complex optimization problems. Some recently developed metaheuristic algorithms perform well, e.g.: red fox optimizer^16^, the flying fox optimizer^17^, polar bear optimization^18^ and the mayfly optimization algorithm^19^. The successful application of these algorithms further demonstrates the effectiveness and potential of metaheuristic algorithms in solving complex optimization problems.

Harmony Search (HS) is an intelligent optimization algorithm proposed by Geem et al. that simulates the process of musical performance^20^. Despite the emergence of a variety of novel metaheuristic algorithms in recent years, the harmony search algorithm still has significant advantages due to its unique mechanism. First, HS collaboratively generates new solutions through memory banks, which can effectively balance global exploration and local exploitation and avoid premature convergence; second, with fewer HS parameters (only harmony memory considering rate (HMCR), pitch adjustment rate (PAR) and Bandwidth (BW)) and low complexity of tuning parameterization, which facilitates practical applications, HS is still a powerful algorithm for solving FJSP problems. However, HS is prone to falling into local optima when solving high-complexity problems, leading many scholars to propose various improvements to the HS algorithm. Orman^21^ introduced a global Harmony Search algorithm that modifies the fine-tuning step size, allowing the new harmony to be influenced by the best harmony in the memory pool. This alleviates the issue of needing to tune the fine-tuning step size. Zhao et al.^22^ introduced a random-key-based effective minimum sequence rule to initialize the harmony memory pool and proposed a hybrid HS algorithm with variable neighborhood search, effectively solving the flow shop scheduling problem. Yuan et al.^23^ combined heuristic and random strategies to initialize the harmony memory pool, ensuring that the initial harmony memory pool has quality and diversity. They proposed a novel hybrid HS algorithm based on an ensemble approach to minimize the manufacturing cycle and solve the flexible job shop scheduling problem. Awadallah et al.^24^ introduced a hybrid harmony search algorithm by introducing the global-best concept from particle swarm optimization to improve the memory consideration operator of the harmony search (HS) algorithm and integrating a hill climbing optimizer (HCO) to enhance its local search capability.This hybrid algorithm effectively addressed highly constrained nurse rostering problems. Abu Doush et al.^25^ introduced a neighborhood heuristic and acoustic search algorithm based on the island model by dividing the population into multiple islands and performing migration operations between the islands, which effectively improves the diversity of the algorithm and solves the problem of scheduling between blocking streams. Abu Doush et al.^26^ combined three neighborhood heuristics (insert-or-swap, insert-invert-block, and full-optimality) to improve the quality of the newly generated harmonies, and proposed an modified harmony search algorithm, which further optimizes the performance of the HS algorithm for the blocking flow shop scheduling problem. Pan et al.^27^ introduced a chaotic harmony search algorithm for solving permutation flow-shop scheduling problems with limited buffers.This algorithm leveraged chaotic sequences to enhance global exploration, combined with an improved improvisation scheme and a chaotic local search algorithm, resulting in effective optimization of scheduling solutions. Wang et al.^28^ introduced a hybrid modified global-best harmony search algorithm for the blocking permutation flow-shop scheduling problem. This algorithm incorporated an insertion neighborhood-based local search and a revised pitch adjustment rule to significantly improve the search efficiency and convergence accuracy under bufferless constraints. Pan et al.^29^ introduced a local-best harmony search algorithm with dynamic sub-harmony memories (DLHS), is proposed to minimize the total weighted earliness and tardiness penalties for a lot-streaming flow shop scheduling problem with equal-size sub-lots. Zhang et al.^30^ proposed an improved mayfly algorithm for the Distributed Flexible Job Shop Scheduling Problem (DFJSP), which effectively improves the algorithm’s global search capability and local optimization performance through discrete mapping, three-layer coding rules, hybrid initialization strategy, active time window decoding strategy, and improved crossover and mutation operations, and provides a new idea for the solution of the DFJSP problem and methods for solving the DFJSP problem.

Although the HS search algorithm performs well in solving complex optimization problems, it still suffers from defects such as premature convergence and parameter statics limitation when dealing with high-dimensional, multi-peak optimization problems. Especially in NP-hard problems such as FJSP, traditional HS algorithms are difficult to effectively balance global exploration and local exploitation, resulting in limited solution quality.

For this reason, this paper proposes a Levy flight-based harmony search algorithm. Introducing the Levy flight.

The main contributions of this paper are as follows:

(1) A Levy flight-based Harmony Search algorithm(LHS) is proposed, which solves the local optimization problem caused by the fixed parameters of the traditional HS algorithm by dynamically and adaptively adjusting the HMCR, PAR and BW;

(2) Introducing the long-tailed jump property of Levy flight to enhance the algorithm’s global exploration ability in the solution space, while improving the local exploitation efficiency through the parameter adaptive mechanism;

(3) The LHS algorithm is applied to the FJSP problem, and the experimental results show that it significantly outperforms the comparative algorithms in terms of solution quality and convergence speed.

The rest of the paper is organized as follows. “Flexible job shop scheduling problem (FJSP)” introduces the flexible job shop scheduling problem, “Levy flight principle” introduces the Levy flight principle, “Harmony search principle” introduces the harmonic algorithm principle, “Proposed methodology: modified harmony search algorithm based on Levy flight” and “Improved LHS algorithm for solving FJSP” describes the proposed algorithm and then it is evaluated and analyzed in “Experimental results and analysis”. Finally, conclusions and future work are provided in “Conclusion and future work”.

## Flexible job shop scheduling problem (FJSP)

Given a set of jobs $\:\text{J}=\{{\text{J}}_{1},{\text{J}}_{2}, \ldots ,{\text{J}}_{\text{n}}\}$, each job $\:{\text{J}}_{\text{i}}$ consists of a sequence of operations $\:{\text{O}}_{\text{i}1},{\text{O}}_{\text{i}2}, \ldots ,{\text{O}}_{\text{i}\text{n}}$, which must be executed one after another in a specified order. The set of machines is $\:\text{U}=\{{\text{M}}_{1},{\text{M}}_{2}, \ldots ,{\text{M}}_{\text{m}}\}$, and each operation $\:{\text{O}}_{\text{i}\text{j}}$ can be processed on a subset of compatible machines $\:{\text{U}}_{\text{i}\text{j}}=\text{U}$. If there exists at least one operation $\:{\text{O}}_{\text{i}\text{j}}$ for which the machine set $\:{\text{U}}_{\text{i}\text{j}}$ is a proper subset of $\:\text{U}$, the job exhibits partial flexibility. In the case of total flexibility, each operation $\:{\text{O}}_{\text{i}\text{j}}$ has its own set of compatible machines $\:{\text{U}}_{\text{i}\text{j}}\subseteq\:\text{U}$. The processing time for each job depends on the machine it is assigned to. Let $\:{\text{d}}_{\text{i}\text{j}\text{k}}$ represent the processing time of operation $\:{\text{O}}_{\text{i}\text{j}}$ on machine $\:{\text{M}}_{\text{k}}$. Preemption is not allowed, meaning each operation must be completed without interruption. Furthermore, each machine can perform at most one operation at a time, and all jobs and machines are available at time zero. The solution to the FJSP consists of two main parts: (1) Assigning each operation to an appropriate machine. (2) Sequencing the operations on each machine to minimize the makespan (the total time required to complete all jobs).

Table 1 illustrates an example of a 3-machine, 3-job problem for better understanding.

**Table 1:** Processing schedule.

Job	Operation	$\:{\text{M}}_{1}$	$\:{\text{M}}_{2}$	$\:{\text{M}}_{3}$	Time of completion	Job weight
	$\:{\text{O}}_{11}$	7	5	10	15	1
$\:{\text{J}}_{1}$	$\:{\text{O}}_{12}$	8	10	5		
	$\:{\text{O}}_{13}$	6	5	8		
	$\:{\text{O}}_{21}$	8	7	15	47	1
$\:{\text{J}}_{2}$	$\:{\text{O}}_{22}$	14	11	-		
	$\:{\text{O}}_{23}$	5	11	8		
	$\:{\text{O}}_{24}$	15	-	12		
	$\:{\text{O}}_{31}$	6	9	5	37	1
$\:{\text{J}}_{3}$	$\:{\text{O}}_{32}$	6	-	7		
	$\:{\text{O}}_{33}$	10	-	9		
	$\:{\text{O}}_{34}$	8	7	10		

In Table 1, assume that job $\:{\text{J}}_{1}$ has three operations, and each operation can be processed on three machines ($\:{\text{M}}_{1},{\text{M}}_{2},{\text{M}}_{3}$). The numbers represent the processing time required by each machine for the corresponding operation. The optimal total time required to process all three operations of job $\:{\text{J}}_{1}$ is 5, and the weight of job $\:{\text{J}}_{1}$ is 1. The descriptions of jobs $\:{\text{J}}_{2}$ and $\:{\text{J}}_{3}$ are similar to that of $\:{\text{J}}_{1}$. A “-” indicates that a particular machine cannot process that operation. The objective function of the FJSP is defined as follows:

1 $$\:{\text{f}=\text{m}\text{i}\text{n}\text{C}}_{\text{m}\text{a}\text{x}},\text{s}.\text{t}.{\text{C}}_{\text{m}\text{a}\text{x}}=\text{m}\text{a}\text{x}({\text{P}}_{\text{j}\text{h}}+{\text{S}}_{\text{j}\text{h}})$$

Where $\:{\text{C}}_{\text{m}\text{a}\text{x}}$ represents the final completion time of the job; $\:{\text{P}}_{\text{j}\text{h}}, {\text{S}}_{\text{j}\text{h}}$ represent the machining time and start time on the h-th process of job $\:\text{j}$, respectively.

## Levy flight principle

Levy flight^31^ is a type of stochastic process with the Markov property, which is of significance in improving intelligent computation. The jump lengths in Levy flight follow a Levy distribution, which has a heavy-tailed asymptotic form: $\:\text{p}\left(\text{x}\right)\sim\frac{1}{{\left|\text{x}\right|}^{\mu\:+1}},0<\mu\:<2$ is the Levy index. The Levy distribution is defined as:

2 $$\:\text{L}(s,\gamma\:,\mu\:)=\left\{\begin{array}{c}\sqrt{(\frac{\gamma\:}{2{\uppi\:}}})exp\left[-\frac{\gamma\:}{2\left(\text{s}-\mu\:\right)}\right]\frac{1}{{\left(\text{s}-\mu\:\right)}^{\frac{3}{2}}},0<\mu\:<s<\infty\:\\\:0,else\end{array}\right.\:$$

where $\:\gamma\:$ is a scale parameter and $\:s$ is the step size.

The random numbers generated by Levy flight consist of two parts: the random direction selection and the step size of the Levy flight. Using the Mantegna algorithm, a balanced Levy stable distribution can be generated. The mathematical expression for the step sizes is:

3 $$\:\text{l}\text{e}\text{v}\text{y}\left(\text{s}\right)=\frac{{\upmu\:}}{{\left|v\right|}^{\frac{1}{{\upbeta\:}}}}$$

Where $\:u$ and $\:v$ are drawn from normal distributions. That is,$\:\mu\:\sim\text{N}(0,{{\upsigma\:}}_{{\upmu\:}}^{2}),v\sim\text{N}\left(\text{0,1}\right)$, and $\:{{\upsigma\:}}_{\mu\:}^{2}=\{\frac{{\Gamma\:}\left(1+{\upbeta\:}\right)\text{s}\text{i}\text{n}\left(\frac{{\uppi\:}{\upbeta\:}}{2}\right)}{{\Gamma\:}\left[(\frac{1+{\upbeta\:})}{2}\right]{\upbeta\:}{2}^{\frac{({\upbeta\:}-1)}{2}}}{\}}^{\frac{1}{{\upbeta\:}}},\:{\upbeta\:}=\frac{3}{2}$, $\:{\Gamma\:}\left(\text{z}\right)$ is the Gamma function.

## Harmony search principle

HS algorithm is a meta-heuristic optimization method based on the process of musical improvisation^20^. The core idea is to simulate the process of musicians adjusting the pitches of different instruments to achieve a state of harmony. In the optimization problem, each “instrument” corresponds to a decision variable, and “harmony” is mapped to the optimal solution of the objective function. HS achieves efficient search by balancing global exploration (randomly generating new solutions) and local exploitation (fine-tuning existing solutions), and is applicable to continuous, discrete and mixed-variable optimization problems. discrete and mixed-variable optimization problems.

The HS algorithm is implemented through the following five main steps^26^.

Usually, in discrete optimization problems with constraints, the objective function $\:f\left(x\right)$ is obtained by mathematical modeling to represent the specific problem:

4 $$\:\text{m}\text{i}\text{n}\text{f}\left(\text{x}\right)\:\:\:\:\:\text{x}\in\:[\:\text{L}\text{B},\text{U}\text{B}]$$

The following constraints are satisfied:

Equality constraints: $$\:{g}_{i}\left(x\right)={c}_{i}$$

Inequality constraints: $$\:{h}_{i}\left(x\right)\le\:{d}_{j}$$

Where $\:x=({x}_{1},{x}_{2}, \ldots ,{x}_{n})$ denotes the solution vector of the $\:n$ decision variables.

**Step 1.** Initialize the required parameters. The parameters of the harmony search algorithm include the following: Harmony Memory Size (HMS), Harmony Memory Considering Rate (HMCR), Pitch Adjusting Rate (PAR) and Bandwidth (BW).

**Step 2.** The initial solution is randomly generated and stored in the Harmony Memory (HM):

5 $$\:HM=\left[\begin{array}{ccc}{x}_{1}^{1}&\:\begin{array}{cc}{x}_{2}^{1}&\:\cdots\:\end{array}&\:{x}_{n}^{1}\\\:\begin{array}{c}{x}_{1}^{2}\\\: \vdots \end{array}\:&\:\begin{array}{cc}{x}_{2}^{2}&\:\cdots\:\\\: \vdots &\:\ddots\:\end{array}&\:\begin{array}{c}{x}_{n}^{1}\\\: \vdots \end{array}\\\:{x}_{1}^{HMS}&\:\begin{array}{cc}{x}_{2}^{HMS}&\:\cdots\:\end{array}&\:{x}_{n}^{HMS}\end{array}\right]\left[\begin{array}{c}\begin{array}{c}f\left({x}^{1}\right)\\\:f\left({x}^{2}\right)\end{array}\\\: \vdots \\\:f\left({x}^{HMS}\right)\end{array}\right]$$

where $\:n$ is the variable dimension and $\:{x}_{i}^{j}$ denotes the i-th variable of the j-th solution.

**Step 3.** Improvise to generate new harmonies. Generate new solutions by the following rules $\:{x}^{{\prime\:}}=({x}_{1}^{{\prime\:}},{x}_{2}^{{\prime\:}},{ \ldots ,x}_{n}^{{\prime\:}})$:

1. Memory bank selection: randomly select variable values from HM with probability HMCR.
2. Random Generation: Generate values randomly with probability 1-HMCR within the variable range.
3. Local fine-tuning: if the variables are from the HM, fine-tune them with probability PAR:

6 $$\:{x}_{i}^{{\prime\:}}\leftarrow\:{x}_{i}^{{\prime\:}}\pm\:bw\bullet\:rand\left(\text{0,1}\right)$$

where $\:bw$ is the bandwidth.

**Step 4.** Updates to the HM. Calculate the fitness value $\:f\left(x{\prime\:}\right)$ of the new solution $\:x{\prime\:}$ and replace it if it is better than the worst solution in the HM.

**Step 5.** Check if the algorithm terminates. Repeat steps (2)-(3) until termination conditions are met (e.g., maximum number of iterations or convergence threshold).

## Proposed methodology: modified harmony search algorithm based on Levy flight

In HS algorithms, the memory considering rate (HMCR), Pitch Adjusting Rate (PAR), and bandwidth (BW) are usually set to fixed values empirically, resulting in a search performance limited by parameter sensitivity. To overcome this problem, various parameter adaptive strategies have been proposed in existing studies. For example, Pan et al.^27^ dynamically adjusted PAR and BW through chaotic perturbations to improve the convergence accuracy of flow shop scheduling, and Yuan et al.^23^ used a heuristic initialization method to optimize HMCR and enhance population diversity. However, these methods often rely on additional operators or complex rules, which increase the computational complexity.

In this paper, we propose a dynamic adaptive adjustment strategy for parameters based on Levy flight. Different from the PSO hybrid method in literature^24^, LHS does not need to introduce an external optimization mechanism, but directly perturbs HMCR and BW through the long-tailed stochasticity of Levy flight (Eqs. 12, 15), so that it promotes global exploration (low HMCR, high PAR) at the beginning of the iteration, and shifts to local exploitation (high HMCR, adaptive BW) at the later stage. This strategy not only simplifies the parameter tuning process but also extends the search space coverage through Lévy flight properties (Eq. 11). This design aligns with the theoretical framework in literature^25^, where parameter self-adaptation is emphasized as the core mechanism for balancing exploration and exploitation.

### Adaptive harmony memory considering rate based on Levy flight

The size of the memory bank fetch probability HMCR value directly affects the entire scope of the algorithm search. The larger HMCR values facilitate the local search of the HS algorithm, and the smaller HMCR values increase the diversity of the harmonic memory bank. Therefore, a dynamic adaptive change HMCR is proposed with the following expression:

7 $$\:{\text{H}\text{M}\text{C}\text{R}}_{\text{t}}={\text{H}\text{M}\text{C}\text{R}}_{\text{m}\text{i}\text{n}}+{\upalpha\:}\left(\frac{{\text{H}\text{M}\text{C}\text{R}}_{\text{m}\text{a}\text{x}}-{\text{H}\text{M}\text{C}\text{R}}_{\text{m}\text{i}\text{n}}}{{\text{T}}_{\text{m}\text{a}\text{x}}}\text{t}\right)+(1-{\upalpha\:})\text{l}\text{e}\text{v}\text{y}\left(\text{s}\right)\:$$

where $\:{\text{H}\text{M}\text{C}\text{R}}_{\text{t}}$ represents the probability of the memory bank fetch at the t-th iteration, $\:{HMCR}_{min}$ represents the minimum value of the probability of the memory bank fetch, $\:{\text{H}\text{M}\text{C}\text{R}}_{\text{m}\text{a}\text{x}}$ represents the maximum value of the probability of the memory bank fetch, $\:\text{l}\text{e}\text{v}\text{y}\left(\text{s}\right)$represents Levy flight random wandering, and $\:{\upalpha\:}$ represents the density function of the normal distribution that satisfies a variance of 1, i.e.,$\:\alpha\:\sim N (\mu\:,1)$. where the expected value of $\:\mu\:$ is determined by the number of iterations of the algorithm. Its improved HMCR dynamic adaptive change curve graph is shown below:

**Fig. 1:**
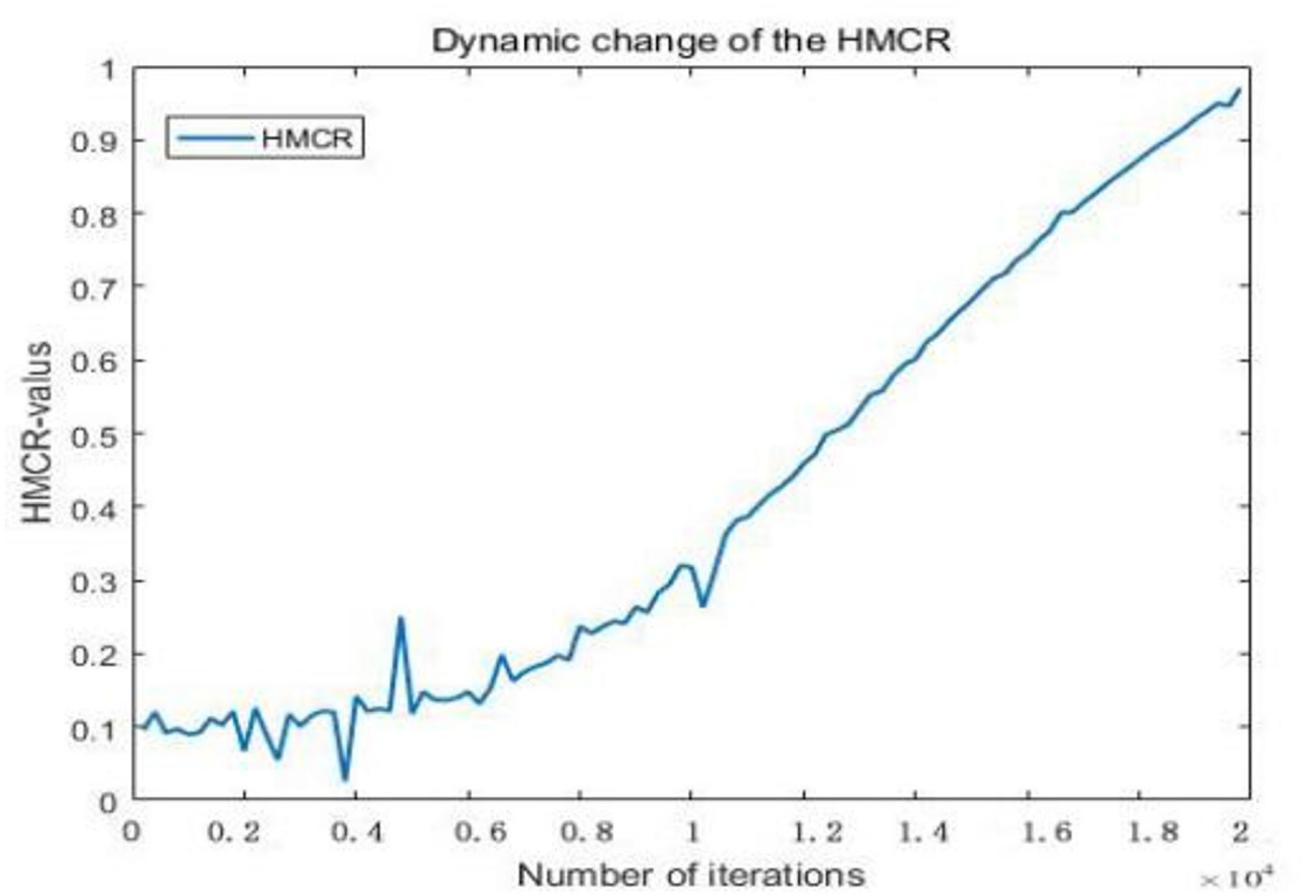
Dynamic change of the $\:{\text{H}\text{M}\text{C}\text{R}}_{\text{t}}$

As seen from Fig. 1, in the early stages of the iterative search, the value of HMCR fluctuates due to the perturbation effect of Levy flight, aking a smaller value that helps increase the diversity of the population and expand the search range. In the later stages of the iterative search, HMCR gradually increases as the number of iterations increases, and the effect of Levy flight disturbance becomes weaker. At this point, the value of HMCR is larger, which helps the algorithm focus on local search and improves the convergence rate.

### Adaptive pitch adjustment rate

A smaller Pitch Adjustment Rate (PAR) enhances the algorithm’s local search capability, while a larger PAR is beneficial for adjusting the search area and expanding the search range. Therefore, an improvement to the PAR is proposed, with the following expression:

8 $$\:{\text{P}\text{A}\text{R}}_{\text{t}}={\text{P}\text{A}\text{R}}_{\text{m}\text{i}\text{n}}+\frac{{\text{P}\text{A}\text{R}}_{\text{m}\text{a}\text{x}}-{\text{P}\text{A}\text{R}}_{\text{m}\text{i}\text{n}}}{\frac{{\uppi\:}}{2}}{\text{tan}}^{-1}\left(\text{t}\right)\:$$

where $\:{\text{P}\text{A}\text{R}}_{\text{t}}$ represents the pitch adjustment probability at the t-th iteration, $\:{\text{P}\text{A}\text{R}}_{\text{m}\text{i}\text{n}}$ and $\:{\text{P}\text{A}\text{R}}_{\text{m}\text{a}\text{x}}$, represents the minimum pitch adjustment probability and the maximum pitch adjustment probability, respectively.

**Fig. 2:**
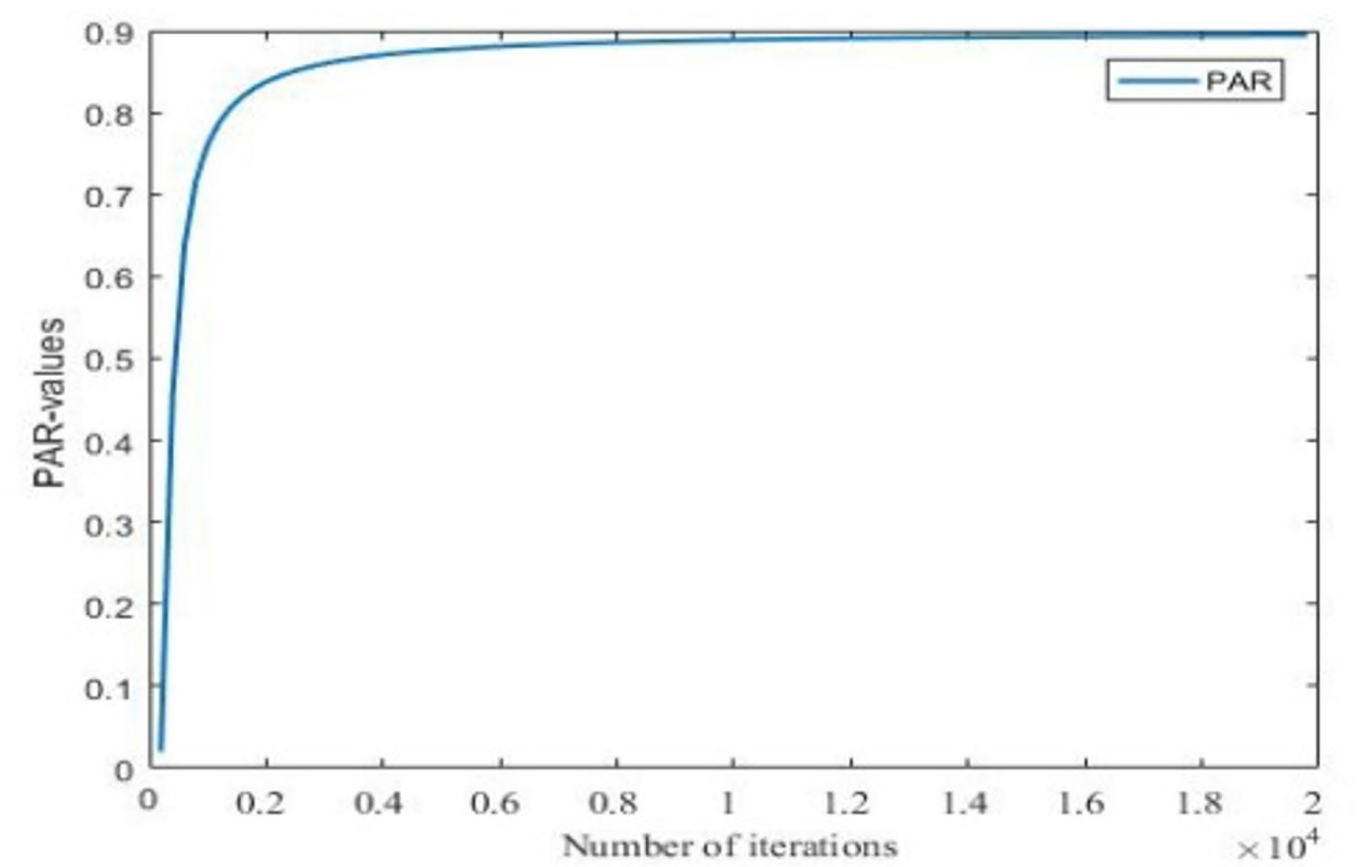
Dynamic change of the $\:{\text{P}\text{A}\text{R}}_{\text{t}}$

As shown in Fig. 2, in the early stages of the iterative search, the PAR value changes rapidly, which helps the algorithm perform local exploration. In the later stages of the search, the PAR value changes more slowly, and the algorithm adjusts the search range using the harmony memory (HM), allowing the LHS to gradually expand the search range across the entire solution vector. This helps the algorithm escape local optima more effectively.

### Adaptive bandwidth based on Levy flight

A larger Bandwidth(BW) helps the Harmony Search algorithm escape from local optima, while a smaller BW favors more refined local search in a confined area. Therefore, Levy flight is used to improve BW, which encourages the algorithm to jump out of local optima and explore other regions of the solution vector, thus enhancing the computational accuracy.The expression for the BW is as follows:

9 $$\:\text{B}\text{W}={\text{a}}_{0} \oplus \text{L}\text{e}\text{v}\text{y}\left(\text{s}\right)\:$$

The expression for generating a new harmony is as follows:

10 $$\:{\text{X}}_{\text{i}}^{\text{n}\text{e}\text{w}}={\text{X}}_{\text{i}}^{\text{b}\text{e}\text{s}\text{t}}\pm\:\text{B}\text{W}\:\:\:\text{i}\in\:(\text{1,2}, \ldots ,\text{n})$$

where $\: \oplus$ is the denotes element-wise multiplication; $\:{\text{a}}_{0}=\sqrt{\text{E}\stackrel{-}{\left(\text{x}\right)}}$ represents the step control quantity, $\:\sqrt{\text{E}\stackrel{-}{\left(\text{x}\right)}}$ represents the open square root of the current population expectation; $\:{\text{X}}_{\text{i}}^{\text{b}\text{e}\text{s}\text{t}}$ represents the optimal harmony for the i-th dimension.

**Fig. 3:**
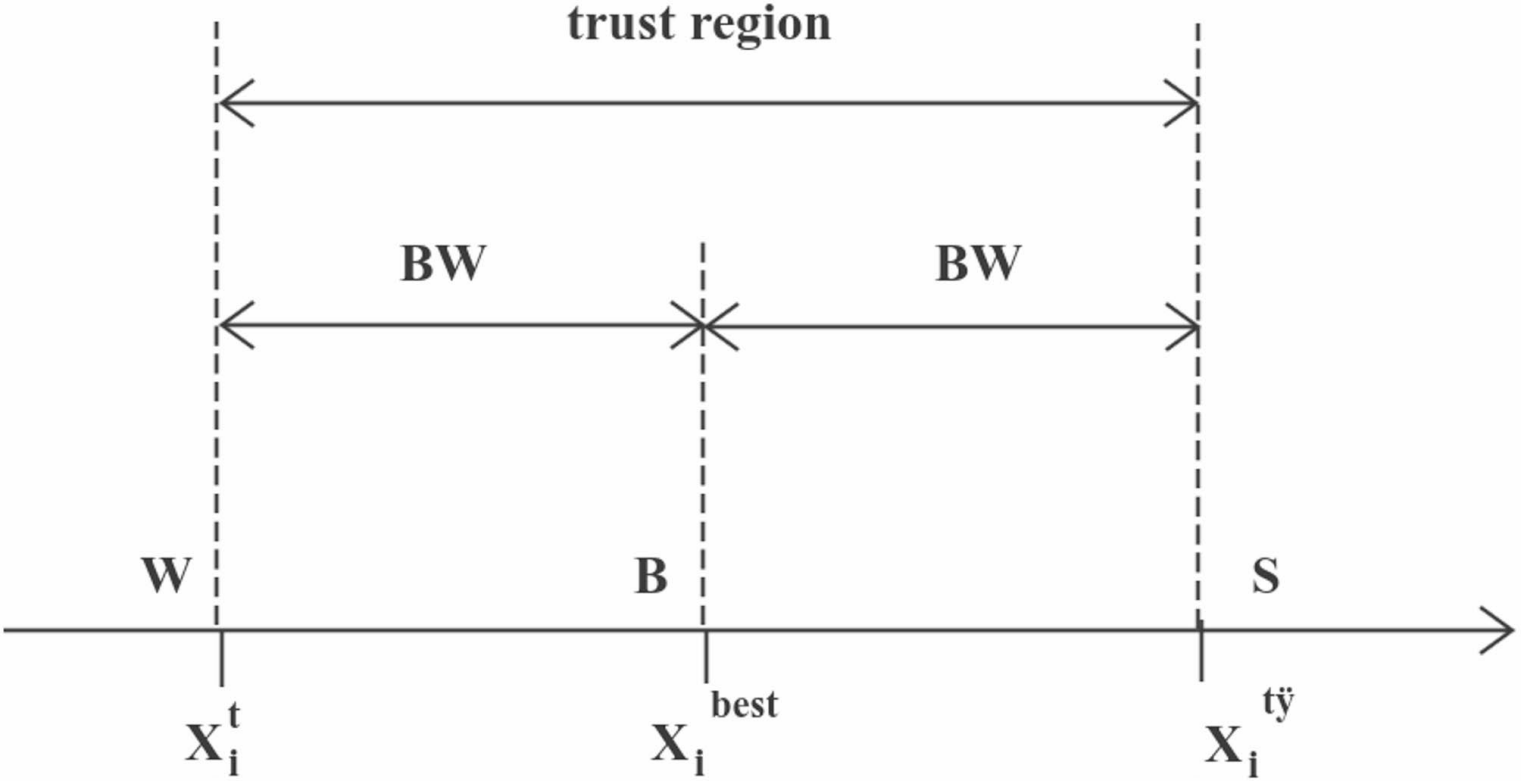
BW principle of dynamic adaptive change.

The principle of BW is shown in Fig. 3 below:

In Fig. 3, $\:\text{W}$ and $\:\text{S}$ are symmetric about point $\:\text{B}$. The distance between points $\:\text{B}$ and $\:\text{S}$ is the defined trust domain, and the distance between $\:{\text{X}}_{\text{i}}^{\text{t}}$ and $\:{\text{X}}_{\text{i}}^{\text{b}\text{e}\text{s}\text{t}}$ is defined as the $\:\text{B}\text{W}$ of the i-th component. In the early stages of the search, the differences between the various harmony vectors are large, resulting in a larger step size and a wider trust region, which facilitates global search. As the search progresses, $\:{\text{X}}_{\text{i}}^{\text{t}}$ moves toward $\:{\text{X}}_{\text{i}}^{\text{b}\text{e}\text{s}\text{t}}$, causing most harmony vectors to converge. At this point, the step size becomes smaller, and the corresponding trust region narrows, promoting more localized search.

**Fig. 4:**
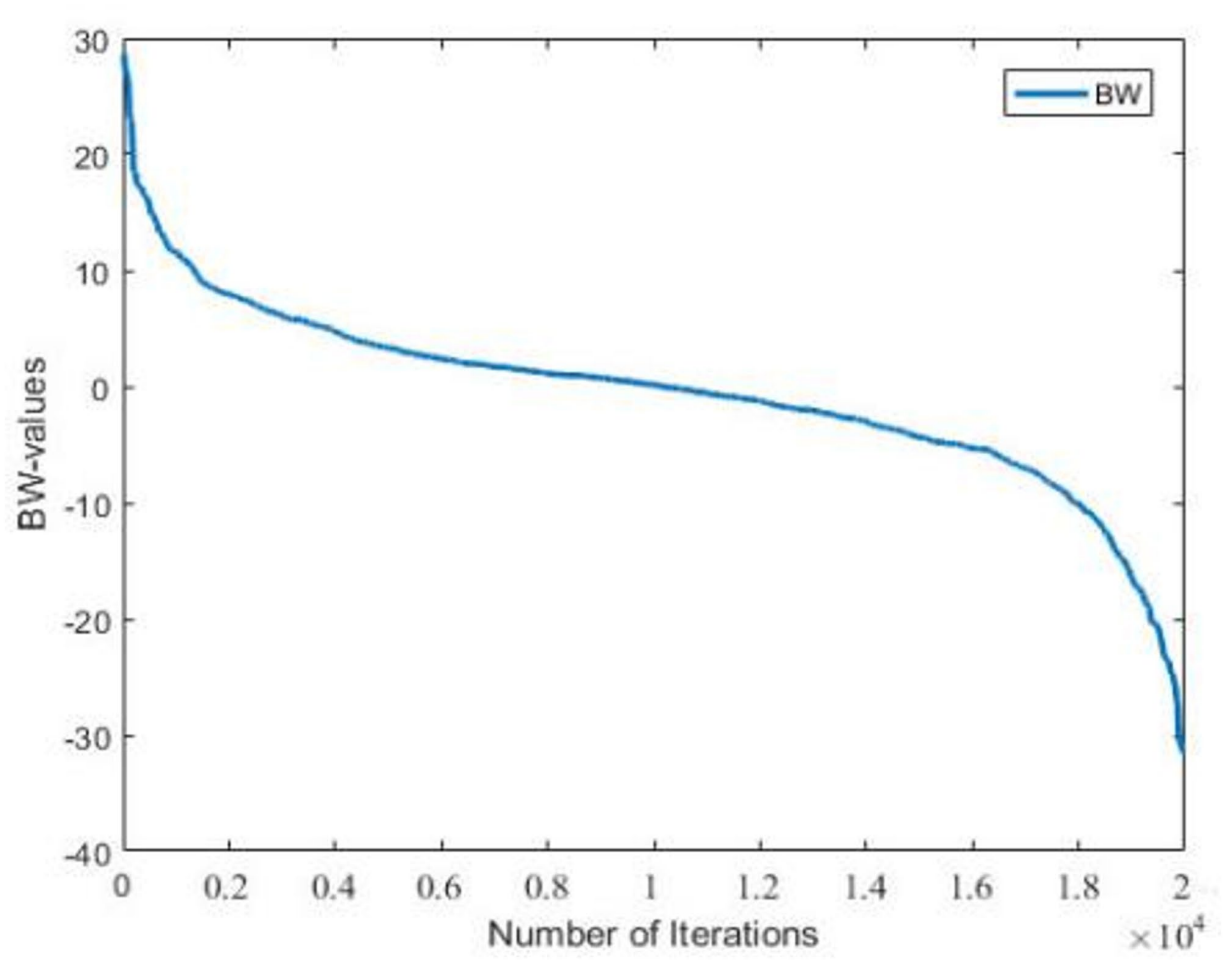
Dynamic change of the BW.

The dynamic adaptive change curve for BW is shown in Fig. 4 below:

The dynamic adjustment strategy of HMCR (Eq. 7) allows the algorithm to expand the search range with lower HMCR values at the beginning of the iteration (Fig. 1) by introducing random perturbations of Levy flights to increase the population diversity, while the HMCR gradually increases with the increase of the number of iterations to strengthen the local search. This design is in line with the principle of “exploration-exploitation” balance of the metaheuristic algorithm, and its effectiveness can be verified indirectly through the convergence analysis (Sect. 5.4): the spectral radius of the iteration matrix in Eq. 19, ρ(M) < 1, indicates that the dynamic adjustment strategy of the algorithm parameters can ensure the convergence of the solution. In addition, the long-tail property of the Levy flight (Eq. 3) further reduces the probability of falling into a local optimum through intermittent large-span jumps.

### Constraint handling in LHS algorithm

For the vast majority of constrained optimization problems, the optimal feasible solution is often located on the boundary. Therefore, the LHS algorithm utilizes a feasible harmony solution $\:{\text{x}}_{\text{f}\text{e}\text{a}}$ to repair infeasible harmony solutions $\:{\text{x}}_{\text{i}\text{n}\text{f}}$ by mapping them to the boundary. In cases where the optimal feasible solution does not lie on the boundary, no further processing is performed by the LHS algorithm.

In practical constraint handling, it is often difficult to directly find the boundary point closest to $\:{\text{x}}_{\text{i}\text{n}\text{f}}$. To address this, the algorithm identifies a boundary point on the line segment connecting the infeasible harmony solution $\:{\text{x}}_{\text{i}\text{n}\text{f}}$ and the feasible harmony solution $\:{\text{x}}_{\text{f}\text{e}\text{a}}$, as referenced in^32^.

#### Lemma

*For any continuous function* $\:\text{g}\left(\text{x}\right)$, *and a pair of points* $\:{\text{x}}_{1}\in\:\left\{\text{x}|\text{g}\left(\text{x}\right)>0\right\}$ *and* $\:{\text{x}}_{2}\in\:\left\{\text{x}|\text{g}\left(\text{x}\right)\le\:0\right\}$, *there always exists a real number* $\:{{\updelta\:}}_{0}\in\:\left[0,1\right]$*such that the following conditions hold*:

11 $$\:\text{g}\left({{\updelta\:}}_{0}{\text{x}}_{1}+\left(1-{{\updelta\:}}_{0}\right){\text{x}}_{2}\right)=0$$

12 $$\:\text{g}\left({{\updelta\:}}_{0}{\text{x}}_{1}+\left(1-{{\updelta\:}}_{0}\right){\text{x}}_{2}\right)\le\:0\:\:\:\:\:\forall\:{\updelta\:}\epsilon\left[0,\right.\left.{{\updelta\:}}_{0}\right)\:$$

#### Theorem

*For any feasible harmony solution* $\:{\text{x}}_{\text{f}\text{e}\text{a}}$ *and infeasible harmony solution* $\:{\text{x}}_{\text{i}\text{n}\text{f}}$, *there always exists a real number* $\:{{\updelta\:}}_{0}\in\:\left[\text{0,1}\right]$ *such that* $\:\:{\updelta\:}\: \epsilon \:\left[0,\right.\left.{{\updelta\:}}_{0}\right),\:\text{g}\left({{\updelta\:}}_{0}{\text{x}}_{\text{i}\text{n}\text{f}}+\left(1-{\updelta\:}\right){\text{x}}_{\text{f}\text{e}\text{a}}\right)\le\:0$, *and the following condition holds for the boundary point*:

13 $$\:{\text{g}}_{\text{i}}\left({\text{x}}_{\text{i}\text{n}\text{f}}\right)>0,\text{i}=\text{1,2}, \ldots ,\text{m}\:$$

14 $$\:{\text{g}}_{\text{i}}\left({\text{x}}_{\text{i}\text{n}\text{f}}\right)\le\:0,\text{i}=\text{m}+\text{1,2}, \ldots ,\text{n}\:$$

15 $$\:{\text{g}}_{\text{i}}\left({\text{x}}_{\text{f}\text{e}\text{a}}\right)\le\:0,\text{i}=\text{1,2}, \ldots ,\text{n}\:$$

Prove that there exists a real number $\:{{\updelta\:}}_{0}\in\:\left[\text{0,1}\right]$ such that $\:{\text{g}}_{\text{i}}\left({\updelta\:}{\text{x}}_{\text{i}\text{n}\text{f}}+\left(1-{\updelta\:}\right){\text{x}}_{\text{f}\text{e}\text{a}}\right)\le\:0,\text{i}=\text{1,2}, \ldots ,\text{n}$ and at least one equality holds.

#### Proof

For the constraints that are not satisfied $\:{\text{g}}_{\text{i}}\left({\text{x}}_{\text{i}\text{n}\text{f}}\right),\text{i}=\text{1,2}, \ldots ,\text{m}$, it follows from the lemma that there exists$\:{{\updelta\:}}_{0}\in\:\left[\text{0,1}\right]$such that $\:{\text{g}}_{\text{i}}\left({{\updelta\:}}_{\text{i}}{\text{x}}_{\text{i}\text{n}\text{f}}+\left(1-{{\updelta\:}}_{\text{i}}\right){\text{x}}_{\text{f}\text{e}\text{a}}\right)=0,\forall\:{\updelta\:}\epsilon\left[0,\right.\left.{{\updelta\:}}_{\text{i}}\right)$and $\:{\text{g}}_{\text{i}}\left({\updelta\:}{\text{x}}_{\text{i}\text{n}\text{f}}+\left(1-{\updelta\:}\right){\text{x}}_{\text{f}\text{e}\text{a}}\right)\le\:0,\forall\:{\updelta\:}\epsilon\left[0,\right.\left.{{\updelta\:}}_{\text{i}}\right)$.

Given a constraint $\:{\text{g}}_{\text{i}}\left({\text{x}}_{\text{i}\text{n}\text{f}}\right),\text{i}=\text{m}+1,\text{m}+2, \ldots ,\text{n}$ that satisfies the condition, if for any $\:{{\updelta\:}}_{\text{i}},\text{i}=\text{m}+1,\text{m}+2, \ldots ,\text{n}$, $\:{\text{g}}_{\text{i}}\left({{\updelta\:}}_{\text{i}}^{{\prime\:}}{\text{x}}_{\text{i}\text{n}\text{f}}+\left(1-{{\updelta\:}}^{{\prime\:}}\right){\text{x}}_{\text{f}\text{e}\text{a}}\right)\le\:0,\text{i}=\text{1,2}, \ldots ,\text{m}$ holds, then let $\:{{\updelta\:}}_{\text{i}}=1$ be defined; otherwise, by the lemma, there exists an $\:{{\updelta\:}}_{\text{i}}\in\:\left[0,\right.\left.{{\updelta\:}}_{\text{i}}^{{\prime\:}}\right)$ such that $\:{\text{g}}_{\text{i}}\left({{\updelta\:}}_{\text{i}}{\text{x}}_{\text{i}\text{n}\text{f}}+\left(1-{{\updelta\:}}_{\text{i}}\right){\text{x}}_{\text{f}\text{e}\text{a}}\right)=0$and $\:\forall\:{\updelta\:} \epsilon \left[0,\right.\left.{{\updelta\:}}_{\text{i}}\right)$, which yields$\:{\text{g}}_{\text{i}}\left({\updelta\:}{\text{x}}_{\text{i}\text{n}\text{f}}+\left(1-{\updelta\:}\right){\text{x}}_{\text{f}\text{e}\text{a}}\right)\le\:0,\forall\:{\updelta\:}\in\:\left[0,\right.\left.{{\updelta\:}}_{\text{i}}\right)$.

From the above, it follows that for all constraints $\:{\text{g}}_{\text{i}}\left({\text{x}}_{\text{i}\text{n}\text{f}}\right),\text{i}=\text{1,2}, \ldots ,\text{m}$, there exists a corresponding $\:{{\updelta\:}}_{\text{i}}\in\:\left[\text{0,1}\right]$such that both $\:{\text{g}}_{\text{i}}\left({{\updelta\:}}_{\text{i}}{\text{x}}_{\text{i}\text{n}\text{f}}+\left(1-{{\updelta\:}}_{\text{i}}\right){\text{x}}_{\text{f}\text{e}\text{a}}\right)=0$ and $\:\forall\:{\updelta\:}\in\:\left[0,\right.\left.{{\updelta\:}}_{\text{i}}\right),{\text{g}}_{\text{i}}\left({\updelta\:}{\text{x}}_{\text{i}\text{n}\text{f}}+\left(1-{\updelta\:}\right){\text{x}}_{\text{f}\text{e}\text{a}}\right)\le\:0$ are satisfied. Consequently, by setting $\:{{\updelta\:}}_{\text{i}}={\text{m}\text{i}\text{n}}_{\text{i}=\text{1,2}, \ldots ,\text{n}}\left\{{{\updelta\:}}_{\text{i}}\right\}$, $\:{{\updelta\:}}_{\text{i}}$ becomes the real number that meets the conditions.

The aforementioned theorem demonstrates that, under the assumption of continuity, there must exist a boundary point on the line segment connecting the infeasible harmony solution and the feasible harmony solution.

### Convergence analysis of the LHS algorithm

Based on the creative process of Harmony Search (HS) algorithm, as documented in literature^33^, the exploration capability of the algorithm is measured using the expected standard deviation of all harmonies within the Harmony Memory (HM). It has been demonstrated that the exploration capability of HS is associated with parameters HMCR, PAR, BW, as well as the upper and lower bounds of variables, LB and UB. The expression for the exploration capability is presented as follows:

16 $$\:\text{E}\left(\text{y}\left(\text{v}\text{a}\text{r}\right)\right)=\left(1-\frac{1}{\text{H}\text{M}\text{S}}\right)\left(\text{H}\text{M}\text{C}\text{R}\text{*}\text{E}\left(\text{x}\left(\text{v}\text{a}\text{r}\right)\right)+\text{H}\text{M}\text{C}\text{R}\left(1-\text{H}\text{M}\text{C}\text{R}\right){\stackrel{-}{\text{x}}}^{2}+/\frac{{\text{B}\text{W}}^{2}}{3}\text{H}\text{M}\text{C}\text{R}\text{*}\text{P}\text{A}\text{R}+\frac{1}{12}\left(\text{H}\text{M}\text{C}\text{R}\right){\left(\text{L}\text{B}-\text{U}\text{B}\right)}^{2}\right)$$

17 $$\:\text{E}\left(\stackrel{-}{\text{y}}\right)=\text{H}\text{M}\text{C}\text{R}\text{*}\text{E}\left(\stackrel{-}{\text{x}}\right)+\frac{1}{2}(1-\text{H}\text{M}\text{C}\text{R})\left(\text{L}\text{B}+\text{U}\text{B}\right)$$

The proof process is detailed in reference^33^. In this paper, the value of BW is $\:\text{B}\text{W}=\sqrt{\text{E}\left(\stackrel{-}{\text{x}}\right)}\text{L}\text{e}\text{v}\text{y}\left(\text{s}\right)$, which leads to:

18 $$\begin{aligned} & \:\text{E}\left(\text{y}\left(\text{v}\text{a}\text{r}\right)\right)=\left(1-\frac{1}{\text{H}\text{M}\text{S}}\right)\bigg(\text{H}\text{M}\text{C}\text{R}\text{*}\text{E}\left(\text{x}\left(\text{v}\text{a}\text{r}\right)\right)\\& \quad +\text{H}\text{M}\text{C}\text{R}\left(1-\text{H}\text{M}\text{C}\text{R}\right){\stackrel{-}{\text{x}}}^{2}+/\frac{{\text{E}\left(\text{x}\left(\text{v}\text{a}\text{r}\right)\right)\text{L}\text{e}\text{v}\text{y}\left(\text{s}\right)}^{2}}{3}\text{H}\text{M}\text{C}\text{R}\text{*}\text{P}\text{A}\text{R}+\frac{1}{12}(1-\text{H}\text{M}\text{C}\text{R}){\left(\text{L}\text{B}-\text{U}\text{B}\right)}^{2}\bigg)\end{aligned}$$

The iterative equation composed of $\:\text{E}\left(\text{y}\left(\text{v}\text{a}\text{r}\right)\right)$ and $\:\text{E}\left(\stackrel{-}{\text{y}}\right)$ is given by:

19 $$\:\left[\begin{array}{c}\text{E}\left(\text{y}\left(\text{v}\text{a}\text{r}\right)\right)\\\:\text{E}\left(\stackrel{-}{\text{y}}\right)\end{array}\right]=\left[\begin{array}{cc}\left(1-\frac{1}{\text{H}\text{M}\text{S}}\right)\text{H}\text{M}\text{C}\text{R}&\:\frac{{\text{L}\text{e}\text{v}\text{y}\left(\text{s}\right)}^{2}}{3}\text{H}\text{M}\text{C}\text{R}\text{*}\text{P}\text{A}\text{R}\\\:0&\:\text{H}\text{M}\text{C}\text{R}\end{array}\right]\text{*}\left[\begin{array}{c}\text{E}\left(\text{x}\left(\text{v}\text{a}\text{r}\right)\right)\\\:\text{E}\left(\stackrel{-}{\text{x}}\right)\end{array}\right]\left(1-\frac{1}{\text{H}\text{M}\text{S}}\right)+/\left[\begin{array}{c}\frac{1}{12}(1-\text{H}\text{M}\text{C}\text{R}){\left(\text{L}\text{B}-\text{U}\text{B}\right)}^{2}\\\:\frac{1}{2}(1-\text{H}\text{M}\text{C}\text{R})\left(\text{L}\text{B}+\text{U}\text{B}\right)\end{array}\right]$$

It can be simplified to Y = MX + B, where the iteration matrix is:

20 $$\:\text{M}=\left[\begin{array}{cc}\left(1-\frac{1}{\text{H}\text{M}\text{S}}\right)\text{H}\text{M}\text{C}\text{R}&\:\frac{{\text{L}\text{e}\text{v}\text{y}\left(\text{s}\right)}^{2}}{3}\text{H}\text{M}\text{C}\text{R}\text{*}\text{P}\text{A}\text{R}\\\:0&\:\text{H}\text{M}\text{C}\text{R}\end{array}\right],\text{B}=\left[\begin{array}{c}\frac{1}{12}(1-\text{H}\text{M}\text{C}\text{R}){\left(\text{L}\text{B}-\text{U}\text{B}\right)}^{2}\\\:\frac{1}{2}(1-\text{H}\text{M}\text{C}\text{R})\left(\text{L}\text{B}+\text{U}\text{B}\right)\end{array}\right]$$

After t iterations, the result is:$\:{\text{Y}}_{\text{t}}={\text{M}}^{\text{t}}\text{x}+({\text{M}}^{\text{t}-1}+{\text{M}}^{\text{t}-2}+ \ldots +{\text{M}}^{2}+\text{M})\text{B}$

The eigenvalues of the iteration matrix $\:\text{M}$ are:$\:{{\uplambda\:}}_{1}=\left(1-\frac{1}{\text{H}\text{M}\text{S}}\right)\text{H}\text{M}\text{C}\text{R}$.

It is known that HMS (Harmony Memory Size) represents the size of the harmony memory bank, with the condition that $\:\text{H}\text{M}\text{S}>1$. Consequently, the spectral radius of the iterative matrix $\:\text{M}$ is denoted as $\:{\uprho\:}\left(\text{M}\right)=\text{H}\text{M}\text{C}\text{R}$. Therefore, $\:{\uprho\:}\left(\text{M}\right)<1$. Hence, $\:\text{E}\left(\text{y}\left(\text{v}\text{a}\text{r}\right)\right)$ and $\:\text{E}\left(\stackrel{-}{\text{y}}\right)$are iteratively convergent, implying that the algorithm exhibits iterative convergence.

## Improved LHS algorithm for solving FJSP

### Encoding and decoding

Zheng and Wang^34^ proposed the following encoding and decoding scheme, where each job is represented by two carriers: the Operation Sequencing Vehicle (OSV) and the Machine Assignment Vehicle (MAV). OSV : This represents the sequence of all operations that satisfy the precedence constraints. MAV : This represents the machine allocation according to the optimal processing time required for the operations in the OSV. The lengths of OSV and MAV are given by: $\:\sum\:_{\text{j}}^{\text{J}}{{\upmu\:}}_{\text{j}}$.

**Fig. 5:**
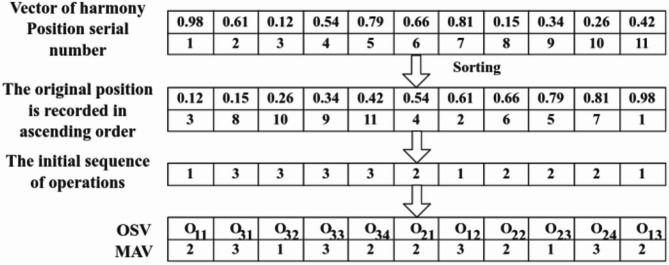
Schematic diagram of encoding.

Based on the dataset presented in Table 1, this coding method is applied to encode the algorithm as proposed. The coding process, as depicted in Fig. 5, is utilized to elucidate the entire coding methodology.

**Fig. 6:**
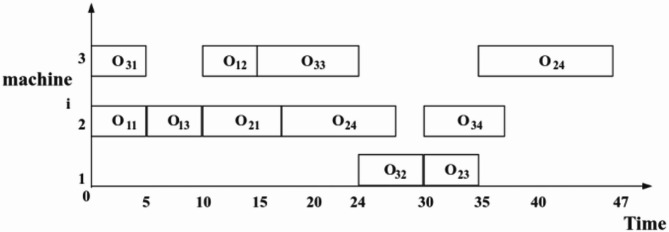
Gantt chart for the entire scheduling activity.

In Fig. 5, assuming that there is a harmonic vector A in continuous space first sort the harmonic vector in ascending order and record the position of each value in the vector to convert the entire harmonic sequence into a discrete sequence that can be read in FJSP. Specifically, we adopt the encoding of OSV and MAV to map the continuous harmonic vectors to discrete process and machine assignments. OSV denotes the sequence of all processes that satisfy the priority constraints, while MAV selects the optimal machine to be assigned based on the order of the processes in OSV. In this way, the LHS algorithm can efficiently optimize in discrete space and ensure its applicability to the discrete nature of FJSP. Utilizing the dataset presented in Table 1, the process is as follows: First, the OSV is employed to sort the transformed harmony vectors for operation sequencing. Subsequently, the MAV is used to allocate machines based on the sorted operations from OSV. The entire scheduling process is shown in the Gantt chart in Fig. 6.

### LHS algorithmic flow for the FJSP problem

**Step 1.** Set the maximum number of iterations and parameters within the LHS algorithm.

**Step 2.** Initialize the HM. Employing an OSV-MAV-based encoding scheme, generate the initial HM for both operations and machines.

**Step 3.** Generate new candidate harmonies. If $\:{{\upgamma\:}}_{1}<\text{H}\text{M}\text{C}\text{R},{{\upgamma\:}}_{1}\in\:\left[\text{0,1}\right]$, the new harmony is generated by selecting a variable value from the HM. Otherwise, the variable value is randomly generated within its corresponding range.

**Step 4.** Perform pitch adjustment on the newly generated harmony. If, use the BW to fine-tune the new harmony. Otherwise, keep the new harmony unchanged.

**Step 5.** Calculate the objective value of the new harmony set. Decode the new harmony after neighborhood transformation, and replace the harmony in the harmony memory with the worst objective value with the newly generated harmony.

**Step 6.** Check if the maximum number of iterations is reached. If the condition is satisfied, proceed to step 7. Otherwise, go back to step 3.

**Step 7.** Terminate the algorithm and output the best solution stored in the HM.

## Experimental results and analysis

The LHS and HS algorithms are tested under $\:8\times\:8$, $\:10\times\:10$, and 10 problem instances proposed by Brandimarte. All test cases are executed in a MATLAB 2016a environment with a Core™@2.50 GHz processor. The parameters for the LHS algorithm are set as follows:$\:\text{H}\text{M}\text{S}=100$, $\:{\text{H}\text{M}\text{C}\text{R}}_{\text{m}\text{i}\text{n}}=0.1$, $\:{\text{H}\text{M}\text{C}\text{R}}_{\text{m}\text{a}\text{x}}=0.9$, $\:{\text{P}\text{A}\text{R}}_{\text{m}\text{i}\text{n}}=0.01$, $\:{\text{P}\text{A}\text{R}}_{\text{m}\text{a}\text{x}}=0.99$. The parameters for the HS algorithm are $\:\text{H}\text{M}\text{C}\text{R}=0.5$, $\:\text{P}\text{A}\text{R}=0.3$, $\:\text{B}\text{W}=0.1$, $\:\text{H}\text{M}\text{S}=100$. Both the LHS and HS algorithms are independently run 10 times to calculate the average value, with $\:{\text{T}}_{\text{m}\text{a}\text{x}}=200$, $\:{\upalpha\:}-\left(\text{5.5,1}\right)$. The obtained results are compared with the Multi-Agent Tabu Search with Local Optimization (MATSLO) and Hybrid Harmony Search (HHS) results from literature^23^, as shown in Table 2.

**Table 2:** Comparison table of running results.

Examples			HS		MATSLO		HHS		LHS	
	n*m	(LB, UB)	Best	Aver	Best	Aver	Best	Aver	Best	Aver
mk01	10*6	(36,42)	44	44.5	40	40.5	40	40	40	40
mk02	10*6	(24,32)	33	33.7	26	27.5	26	26	26	26.2
mk03	15*8	(204,211)	212	214.4	204	205	204	204	204	204
mk04	15*8	(48,81)	74	74.5	60	61.7	60	50	60	60
mk05	15*4	(168,186)	181	181	173	173	172	172.8	171	172.1
mk06	10*15	(33,86)	58	63.1	62	63.5	58	59.1	55	58.6
mk07	20*5	(133,157)	153	154	148	148.6	139	139.5	141	141.2
mk08	20*10	(523)	560	562	523	540.5	523	523	523	523
mk09	20*10	(299,369)	350	351.7	315	315.4	307	307	301	301.7
mk10	20*15	(165,296)	260	262	220	220.4	205	211.1	205	208
8*8	8*8	(14,NA)	18	18.5	14	15	-	-	14	14.5
10*10	10*10	(7,NA)	13	13	7	7.5	-	-	7	7

Table 2 shows the optimal and average values of the LHS algorithm as well as the HS, MATSLO and HHS algorithms for solving the FJSP problem. By comparing the average and optimal values (when the optimal values are the same), it can be seen that the smaller the value, the better the algorithm is optimized. Overall, the average and optimal values of the LHS algorithm are better than or at least comparable to those of HS and MATSLO in most of the instances, indicating a significant optimization performance improvement in solving the FJSP problem. Compared to the HHS algorithm, the LHS also exhibits better or comparable performance in most instances, especially in large-scale instances where the optimization advantage of the LHS is more obvious.

As an example, the mean value of LHS in mk01 (10 × 6) is 40, which is the same as HHS and better than HS (44.5) and MATSLO (40.5), indicating that LHS is able to find the globally optimal solution quickly and maintains the stability in small-scale instances. In mk06 (10 × 15), the average value of LHS is 58.6, which is better than HS (63.1), MATSLO (63.5) and HHS (59.1), which indicates that LHS can effectively avoid local optimality and find better solutions in medium-sized instances. In the large-scale instance mk09 (20 × 10), the average value of LHS is 301.7, which is better than HS (351.7), MATSLO (315.4), and HHS (307), indicating that LHS significantly improves the optimization performance through dynamic parameter tuning based on Levy flight. Similarly, in mk10 (20 × 15), LHS has a mean value of 208, which is superior to HS (262), MATSLO (220.4) and HHS (211.1), further demonstrating its superiority in large-scale problems.

The improved optimization performance of the LHS algorithm is mainly due to the dynamic parameter tuning strategy based on Levy flights.The long-tailed distribution characteristic of Levy flights significantly enhances the algorithm’s global exploration ability, which enables it to skip the local optimal region and expand the search scope at the beginning of the iteration. As the iterations proceed, the dynamically adjusted HMCR, PAR and BW parameters based on Levy flights further balance the global exploration and local exploitation, improving the convergence speed and accuracy of the algorithm. In addition, the improved constraint handling method and OSV-MAV coding method ensure the efficient optimization of the algorithm in discrete space to satisfy the discrete characteristics of FJSP.

Therefore, in terms of the overall effect, the LHS algorithm has good feasibility in solving FJSP, and the introduction of Levy flight mechanism in the standard harmonic search algorithm and the adaptive improvement of parameters are effective and can significantly improve the optimization performance of the algorithm.

In addition, the application of LHS algorithm in the FJSP example and the scheduling of the process processing machines in the mk01 scheduling optimization process are visually demonstrated through the Gantt chart and the convergence curve graph, which further verifies its optimization performance.

**Fig. 7:**
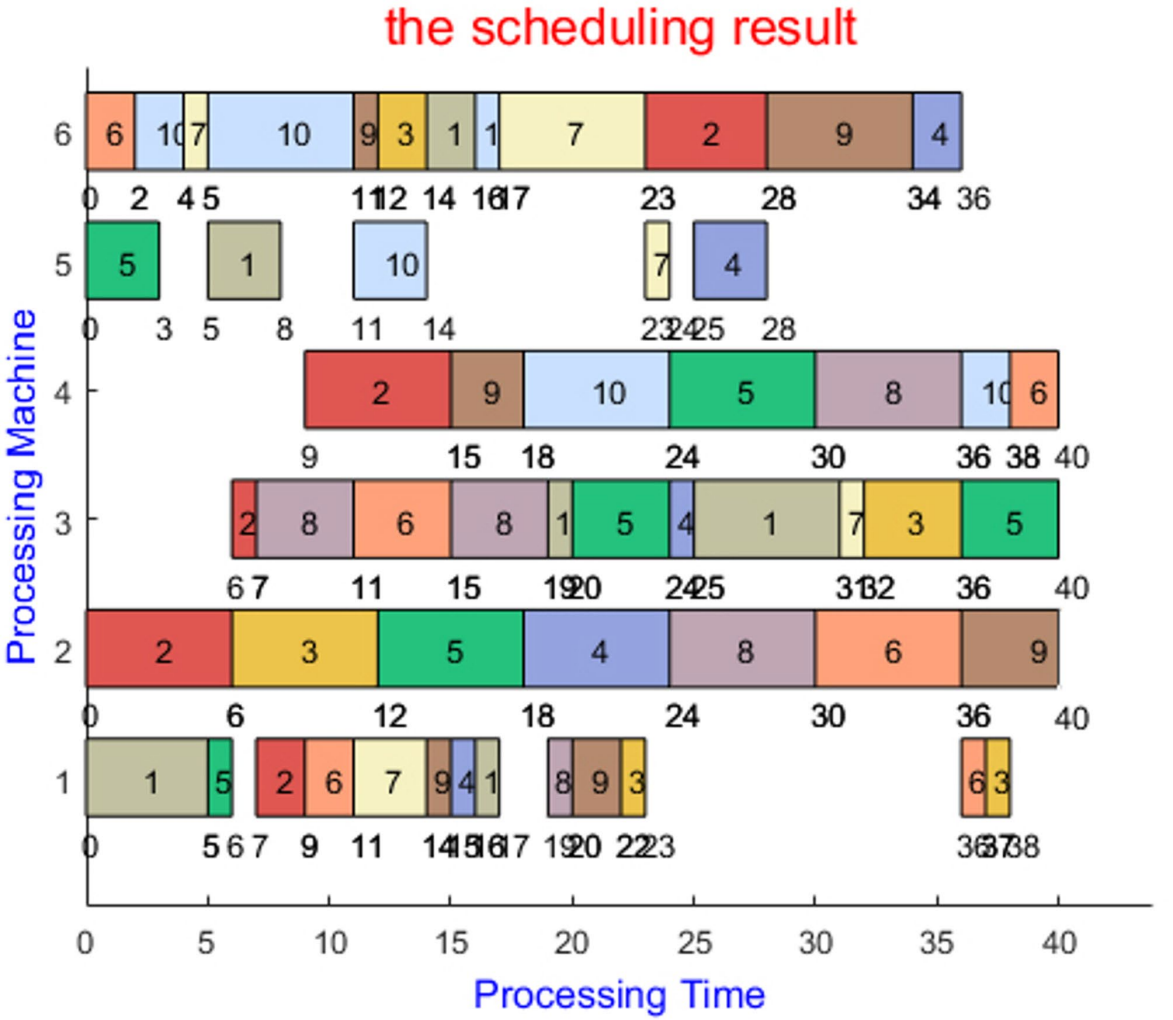
Gantt chart of mk01’s optimal solution scheduling.

**Fig. 8:**
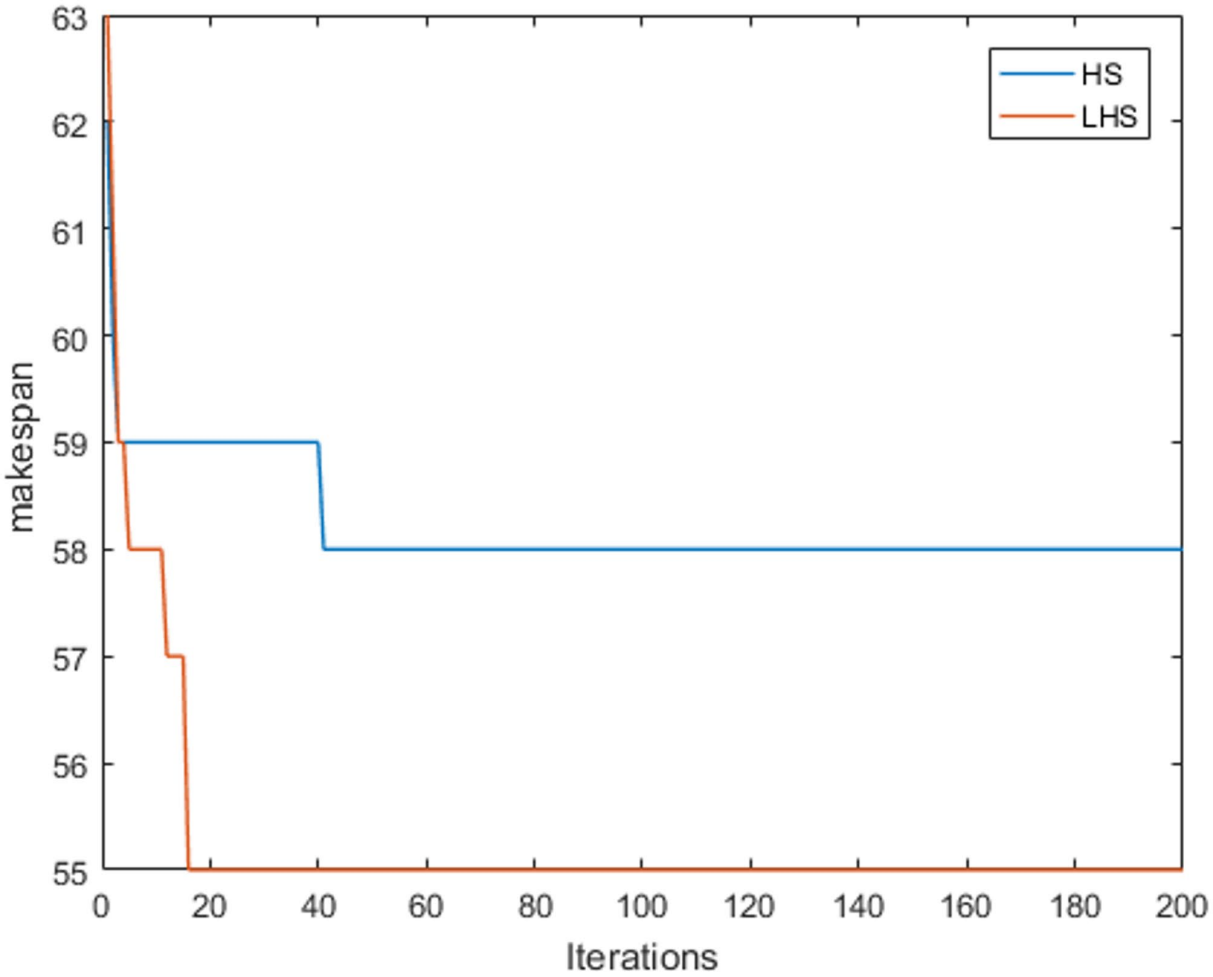
Convergence curve of mk06.

Figure 7 illustrates the Gantt chart of the LHS algorithm during the optimization process of instance mk01, while Fig. 8 depicts the convergence curve of the LHS algorithm during the optimization process of instance mk06.

### Convergence analysis

Figure 8 shows that the objective function value of the LHS algorithm decreases rapidly at the beginning of the iteration and stabilizes in subsequent iterations. The HS algorithm, on the other hand, requires more iterations to gradually approach the optimal solution. This indicates that the LHS algorithm is able to converge quickly and has better convergence properties. This fast convergence ability is mainly attributed to the combination of the Levy flight mechanism and the dynamic parameter tuning strategy, which enables the algorithm to achieve an effective balance between global exploration and local exploitation.

The HS algorithm significantly enhances the global exploration capability while ensuring convergence by dynamically tuning the HMCR, PAR, and BW parameters in combination with the long-tailed distribution property of Levy flight. The dynamic adjustment strategy enables the algorithm to quickly jump out of the local optimal solution and enter into a wider search space at the beginning of the iteration; while at the later stage of the iteration, the gradually decreasing BW values and increasing HMCR and PAR values focus on the local optimization to achieve rapid convergence.The introduction of Levy flight mechanism further avoids the algorithm from falling into the local optimum in the complex optimization problem, which provides a strong guarantee for the convergence.

### Diversity analysis

To quantify the performance of LHS in terms of population diversity, we used the concept of population moisture (Shannon Entropy)^35^. Population moisture is a measure of population diversity that is based on the concept of entropy in information theory and reflects the uniformity of the distribution of individuals in a population.

The diversity of the population is measured by calculating the entropy value for each operational position. The specific steps are as follows:

1. Extraction of population manipulation sequences

Let HM be $\:\text{H}\left(\text{X}\right)=\{{\text{h}}_{1},{\text{h}}_{2}, \ldots ,{\text{h}}_{\text{N}}\}$, where $\:\text{N}$ is the size of the Harmony Memory, and each harmony vector $\:{\text{h}}_{\text{i}}$ contains a sequence of operations. We extract all the operations as sequences, denoted as $\:\text{M}\text{A}\text{V}\text{S}=\{{\text{m}}_{1},{\text{m}}_{2}, \ldots {\text{m}}_{\text{N}}\}$, where $\:{\text{m}}_{\text{i}}$ denotes the operation sequence of the i-th harmonic vector.

2. Calculate the entropy value for each operation position

For each operation position $\:\text{i}\in\:(1\le\:\text{i}\le\:\text{M})$, its entropy value is defined as:

21 $$\:{{p}_{j}^{\left(i\right)}=\frac{{n}_{j}^{\left(i\right)}}{N},H}_{i}=-\sum\limits_{j\in\:{S}_{i}}^{n}{p}_{j}^{\left(i\right)}log\left({p}_{j}^{\left(i\right)}\right)$$

where $\:N$, denotes the number of harmony vectors in the harmony memory. $\:M$ denotes the length of each sequence of operations. $\:{S}_{i}$ denotes the set of all possible operations at the $\:i$-th operation position. $\:{\text{n}}_{\text{j}}^{\left(\text{i}\right)}$ denotes the number of occurrences of operation $\:j$ at the $\:i$-th operation position. $\:{\text{p}}_{\text{j}}^{\left(\text{i}\right)}$ denotes the probability that operation $\:j$ is at the $\:i$-th operation position. $\:log$ is the natural logarithm. The higher the entropy value, the more diversified the operations at that position; the lower the entropy value, the more concentrated the operations at that position.

3. Calculate the average entropy of the population

The entropy values of all operational positions were summed and averaged to obtain a diversity measure for the entire population:

22 $$\:\text{H}\left(\text{t}\right)=\frac{1}{\text{M}}\sum\limits_{\text{i}=1}^{\text{M}}{H}_{i}$$

In this study, we calculated the average population moisture of the populations after each iteration and normalized it for comparison purposes. The normalization was done by dividing the population moisture value calculated for each iteration by the population moisture value at the beginning of the iteration. In this way, we can obtain a normalized diversity value between 0 and 1, where 1 indicates maximum diversity and 0 indicates minimum diversity.

**Fig. 9:**
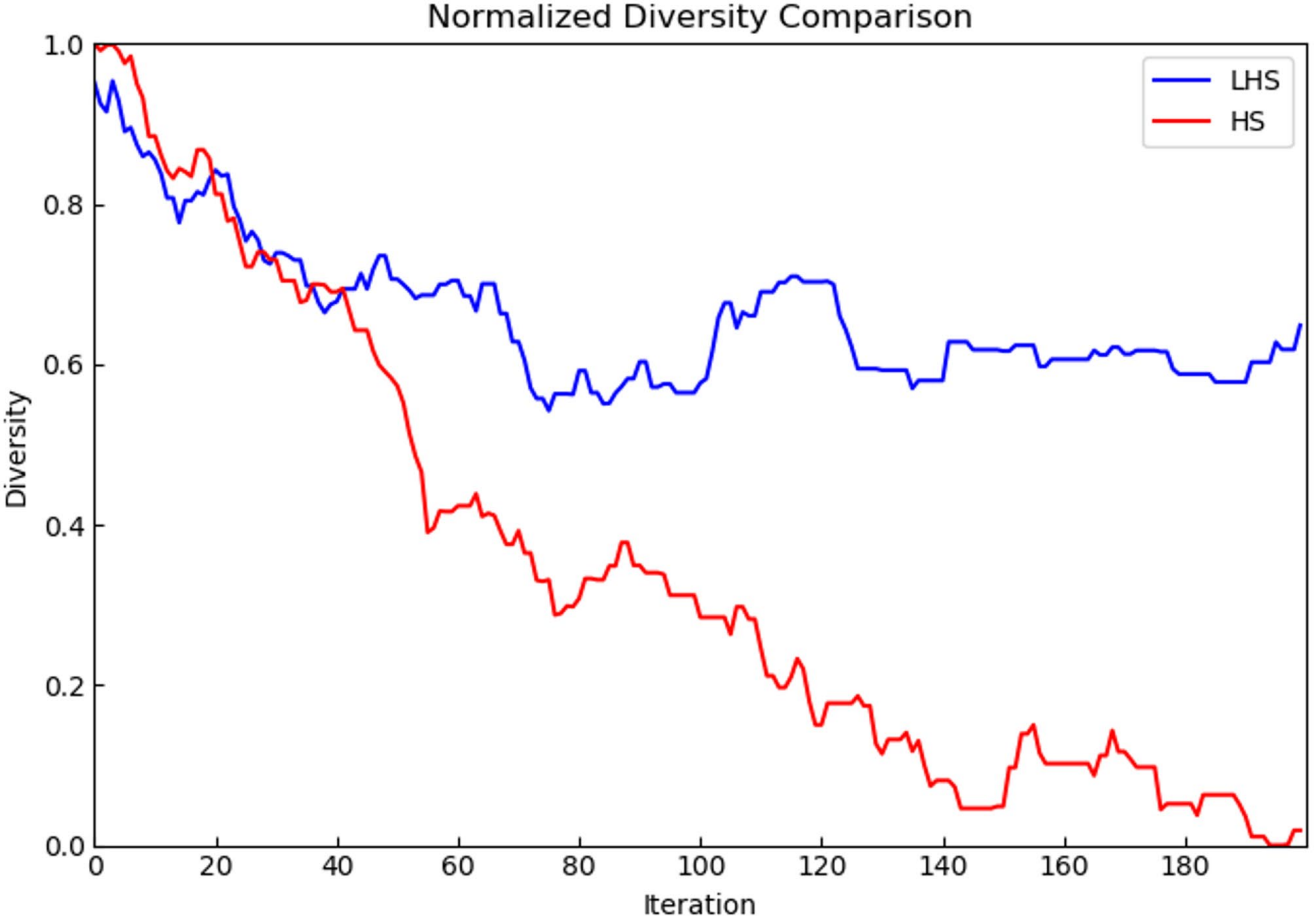
Diversity profile of mk06.

It can be observed from Fig. 9 that although the population diversity of both algorithms decreases with the number of iterations in the early stage, the LHS algorithm still maintains a higher level of diversity in the middle and late search process. This higher diversity helps the algorithms to avoid premature convergence and to find optimal solutions in a wider search space.

The ability of the LHS algorithm to maintain a higher level of population diversity is mainly due to the dynamic parameter tuning strategy based on Levy flights. The dynamic parameter tuning strategy utilizes smaller HMCR values, PAR values and larger BW values at the beginning of the iteration, which promotes population diversity and allows the algorithm to explore within a larger search space. As the iterations progressed, progressively larger HMCR values, PAR values and reduced BW values allowed the algorithm to search localized regions more finely, while maintaining some global search capability. This strategy ensures the algorithm’s diversity during the search process.The Levy flight mechanism, on the other hand, increases the diversity of the population by introducing long jumps, preventing the algorithm from converging to a local optimum prematurely.

Overall, the results in Fig. 9 further validate the effectiveness of the LHS algorithm in increasing population diversity and enhancing global search capability.

### Statistical tests analysis

In order to further validate the performance benefits of the proposed Lévy flight-based harmonic search algorithm (LHS) in the flexible job shop scheduling problem (FJSP). We performed a paired-sample statistical test on the average results of the LHS algorithm against the other compared algorithms (HS, MATSLO, and HHS), and Table 3 shows the test results.

**Table 3:** Comparison table of statistical test results.

	LHS vs. HS	LHS vs. MATSLO	LHS vs. HHS
t-statistic	3.3794	3.0764	-0.1839
p-value	0.0081	0.0132	0.8581

In Table 3, the LHS algorithm has a significant performance advantage over the HS and MATSLO algorithms (p-value less than 0.05 in all cases), indicating that the LHS algorithm is able to obtain a better solution when solving FJSP. While compared to the HHS algorithm, the LHS performs slightly better in some instances, but the p-value is greater than 0.05, which indicates that the two are closer in performance. Overall, the LHS algorithm shows good optimization performance and convergence properties in solving the FJSP problem.

## Conclusion and future work

In this paper, a harmony search algorithm based on Levy flight is proposed, which improves the deficiency of the harmony search algorithm that is prone to fall into the local optimal solution by dynamically and adaptively adjusting the HMCR, PAR and BW parameter according to the previous experience. The parameters of the algorithm are improved by introducing Levy flight, which effectively balances the global search capability and local search capability, and is applied to the FJSP. We tested the proposed method using 10 instances presented by 8*8,10*10 and Brandimarte. The experimental results show that this LHS algorithm obtains better solutions with better convergence properties than other optimization algorithms, and good results are obtained in solving the FJSP.

Statistical test results further validate the performance advantages of the LHS algorithm. Compared with the HS and MATSLO algorithms, the LHS is statistically significantly better than these two algorithms, indicating its higher optimization efficiency in solving the FJSP problem. Although there is no statistically significant difference between the LHS and HHS algorithms, the LHS performs better in certain complex instances with comparable overall performance. This finding is consistent with the results of existing studies on the application of intelligent optimization algorithms to scheduling problems, and further demonstrates the effectiveness of the introduction of the Levy flight mechanism in improving the harmonic search algorithm.

Future research can be carried out in the following aspects:

Incorporating real-time scheduling scenarios, mixing LHS with other algorithms, or exploring its application in dynamic or uncertain environments.

## Data Availability

The original contributions presented in this study are included in the article/supplementary material. Further inquiries can be directed to the corresponding author(s).
